# Early Onset Multiple Primary Tumors in Atypical Presentation of Cowden Syndrome Identified by Whole-Exome-Sequencing

**DOI:** 10.3389/fgene.2018.00353

**Published:** 2018-08-31

**Authors:** Mathias Cavaillé, Flora Ponelle-Chachuat, Nancy Uhrhammer, Sandrine Viala, Mathilde Gay-Bellile, Maud Privat, Yannick Bidet, Yves-Jean Bignon

**Affiliations:** ^1^INSERM, U1240 Imagerie Moléculaire et Stratégies Théranostiques, Université Clermont Auvergne, Clermont-Ferrand, France; ^2^Département d’Oncogénétique, Centre Jean Perrin, Clermont-Ferrand, France

**Keywords:** Cowden syndrome, *PTEN*, CEACAM1, MIB2, melanoma, renal carcinoma, MALT lymphoma, whole-exome-sequencing

## Abstract

A family with an aggregation of rare early onset multiple primary tumors has been managed in our oncogenetics department: the proband developed four early onset carcinomas between ages 31 and 33 years, including acral melanoma, bilateral clear cell renal carcinoma (RC), and follicular variant of papillary thyroid carcinoma. The proband’s parent developed orbital lymphoma and small intestine mucosa-associated lymphoid tissue (MALT) lymphoma between 40 and 50 years old. Whole-exome-sequencing (WES) of the nuclear family (proband, parents, and sibling) identified in the proband a *de novo* deleterious heterozygous mutation c.1003C > T (p.Arg335^∗^) in the phosphatase and tensin homolog (*PTEN*) gene. Furthermore, WES allowed analysis of the nuclear family’s genetic background, and identified deleterious variants in two candidate modifier genes: *CEACAM1* and *MIB2*. *CEACAM1*, a tumor suppressor gene, presents loss of expression in clear cell RC and is involved in proliferation of B cells. It could explain in part the phenotype of proband’s parent and the occurrence of clear cell RC in the proband. Deleterious mutations in the *MIB2* gene are associated with melanoma invasion, and could explain the occurrence of melanoma in the proband. Cowden syndrome is a hereditary autosomal dominant disorder associated with increased risk of muco-cutaneous features, hamartomatous tumors, and cancer. This atypical presentation, including absence of muco-cutaneous lesions, four primary early onset tumors and bilateral clear cell RC, has not been described before. This encourages including the *PTEN* gene in panel testing in the context of early onset RC, whatever the histological subtype. Further studies are required to determine the implication of *CEACAM1* and *MIB2* in the severity of Cowden syndrome in our proband and occurrence of early onset MALT lymphoma in a parent.

## Background

PTEN hamartoma syndrome (PHTS) is an autosomal dominant disorder characterized by deleterious mutation in the tumor suppressor gene phosphatase and tensin homolog (*PTEN*) gene (Blumenthal and Dennis, 2008). The clinical presentation is heterogeneous, including Cowden syndrome (CS) (Pilarski, 2009), Bannayan-Riley-Ruvalcaba syndrome (Lachlan et al., 2007),Lhermitte–Duclos disease (Zhou et al., 2003), Segmental outgrowth-lipomatosis-arteriovenous malformation-epidermal nevus (SOLAMEN) syndrome (Caux et al., 2007), and autism-macrocephaly syndrome (Butler et al., 2005).

Cowden syndrome is the most frequent of these entities, with a prevalence of 1 in 250,000 individuals (Nelen et al., 1999), and penetrance of up to 90% in the second decade (Ngeow and Eng, 2015). CS is associated with increased risk of muco-cutaneous features, hamartomatous tumors and cancer, defined by diagnostic and testing criteria (Saslow et al., 2012; Pilarski et al., 2013) (**Table 1**). Muco-cutaneous lesions including trichilemmoma, acral keratosis and oral papillomatosis, plus macrocephaly are the most frequent features, described in more than 90% of cases after the third decade (Nosé, 2016).

**Table 1:** Testing and clinical diagnosis criteria.

CS/PHTS testing criteria
Individual from a family with a known PTEN mutation
Individual meeting clinical diagnosis criteria for CS/PHTS
Individual with personal history of:
- BRRS or Lhermitte–Duclos disease or Autism spectrum disorder and macrocephaly or
- Two or more biopsy-proven trichilemmomas or
- two or more major criteria (one must be macrocephaly) or
- Three major criteria without macrocephaly or
- One major and > 3 minor criteria or
- >4 criteria
At-risk individual with a relative with a clinical diagnosis of CS/PHTS or BRRS for whom testing has not been performed (At-risk: any one major criterion or two minor criteria)

**Clinical diagnosis criteria for CS/PHTS**

Major criteria:
Breast cancer
Endometrial cancer
Follicular thyroid cancer
Multiple gastro-intestinal hamartomas or ganglioneuromas
Macrocephaly
Macular pigmentation of gland penis
Mucocutaneous lesions (one biopsy-proven trichilemmoma, multiple palmoplantar keratosis, multiple or extensive oral mucosal papillomatosis, multiple cutaneous facial papules)

Minor criteria:
Autism spectrum disorder
Colon cancer
>3 esophageal glycogenic acanthoses
Lipomas
Intellectual disability (QI < 75)
Papillary or follicular variant of papillary thyroid cancer
Thyroid structural lesions
Renal cell carcinoma
Single gastro-intestinal hamartoma or ganglioneuroma
Testicular lipomatosis
Vascular anomalies
Operational diagnosis in an individual (either of the following):
(1) Three or more major criteria, but one must include macrocephaly, Lhermitte–Duclos disease, or gastrointestinal hamartomas; or
(2) Two major and three minor criteria.
Operational diagnosis in a family where one individual meets revised PTEN hamartoma tumor syndrome clinical diagnostic criteria or has a PTEN mutation:
(1) Any two major criteria with or without minor criteria; or
(2) One major and two minor criteria; or
(3) Three minor criteria.


*Testing criteria (Version 2.2016) are defined by National Comprehensive Cancer Network.*

*Clinical diagnosis criteria have been proposed and revised by Pilarski et al. (2013).*

Cowden syndrome also predisposes to cancer, with a high lifetime risk of developing breast or endometrial cancer in women, and thyroid cancer in both sexes. Other cancer risks have recently been identified, including renal carcinoma (RC), gastro-intestinal cancer, and melanoma. RC in CS is predominately of the papillary and chromophobe type, beginning around 40–50 years, while the lifetime risk is unclear. Melanoma is uncommon, with a lifetime risk about 6% (Tan et al., 2012; Bubien et al., 2013). Second cancers are frequent in these patients, but less than 5% develop a third cancer (Ngeow et al., 2014).

Here we described an atypical clinical presentation of CS, which did not meet diagnostic or testing criteria, in a patient who developed four primary early onset carcinomas (one melanoma, two clear cell renal carcinomas, and a follicular variant of papillary thyroid carcinoma), without muco-cutaneous lesions. A heterozygous deleterious mutation in *PTEN* was identified by whole exome-sequencing (WES).

## Case Presentation

The proband presented with a suspect nevus localized on the inner edge of the left foot. Excision revealed acral lentiginous melanoma (Breslow index at 0.41 mm, Clark level II). Extension assessment, which included cerebro-thoraco-abdomino-pelvic computed tomography and inguinal ultrasound, found no secondary lesions, but a small cystic lesion on the upper pole of the left kidney. No complementary treatment was given.

Control tomography 3 months later showed an increase in size of the renal lesion, associated with irregular contours and a heterogeneous aspect, needing expanded nephrectomy. Pathological examination diagnosed clear cell RC, Fuhrman grade II, pT1aNxMx. No complementary treatment was given.

The patient also developed Hashimoto thyroiditis with multinodular goiter of which three nodules measured > 15 mm. Total thyroidectomy revealed follicular variant of papillary carcinoma, pT1NxMx. The patient was treated with Iodine 131.

During surveillance of primary left RC by computed tomography, a cystic lesion of the right kidney with peripheral contrast enhancement was observed, confirmed by magnetic resonance imaging. Gastroendoscopy was performed, removing a single non-inflammatory gastric polyp. No oesophageal glycogenic acanthosis was observed. A partial right nephrectomy was performed. Pathological examination revealed clear cell RC, Furhman grade III, pT1aNxMx. No complementary treatment was given.

Examination revealed no other medical antecedents. Identification of risk factors identified moderate obesity [Body Mass Index (BMI) at 33]. The patient had never smoked or been exposed to irradiation. Physical examination by the dermatologist, surgeon and oncogeneticist identified no additional lesions, in particular no cutaneous lesions evocative of CS (trichilemmoma, palmoplantar keratoses, oral papillomatosis, and facial papules). The cranial perimeter was unknown. Brain imaging revealed no Lhermitte–Duclos disease.

### Family History

**FIGURE 1:**
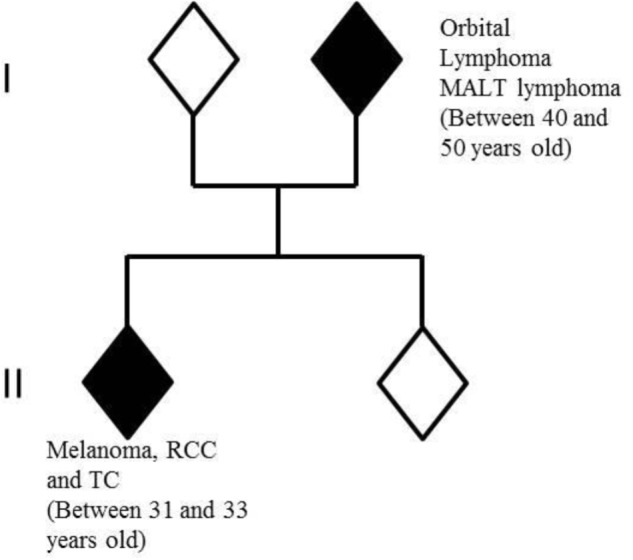
Members of nuclear family analyzed by whole-exome-sequencing. In white: healthy individuals; in black: individuals with cancers. RCC, renal cell carcinoma; TC, thyroid carcinoma.

One of the proband’s parents had developed two distinct rare cancers: orbital lymphoma and small intestine mucosa-associated lymphoid tissue (MALT) lymphoma between 40 and 50 years old. No other familial history of cancer or other medical problems were observed (**Figure 1**).

### Genetic Exploration

Because of multiple RCs, genetic analysis of *VHL* was performed in 2009: no qualitative or quantitative mutation was identified. Other genes involved in hereditary RC (*MET*, *FH*, *FLCN*) were not explored because the histological type of RC and examination did not correspond.

## Laboratory Investigation

Whole-exome-sequencing of the nuclear family was performed in order to identify constitutional causal mutation(s) for this clinical presentation.

### Samples

The nuclear family included the proband, sibling, and parents. Each patient signed informed consent for genetic diagnosis and research for hereditary disease. The local ethics committee at Clermont-Ferrand did not oppose the publication of this case report without patient specific consent. Three samples from the proband’s thyroid tumor were included (cancer cells: 70–100%). Melanoma and renal samples were not available. DNA extraction of tumor samples was performed using Maxwell 16 FFPE Plus LEV kit from formalin-fixed paraffin-embedded blocks.

### Whole-Exome-Sequencing

Whole-exome-sequencing was performed on four family members. DNA was extracted from peripheral blood using QIAamp DNA macrokit (Qiagen). Sonic fragmentation was performed on a Bioruptor instrument (Diagencode). Kapa library preparation and SeqCap EZ MedExome kits (Roche) were used for library preparation and capture. Sequencing was performed using NextSeq 500/550 High Output v2 kit (300 cycles) on a NextSeq 500 instrument (Illumina). All steps were performed following providers’ guidelines.

#### Bioinformatics Analysis

De-multiplexing was performed using bcl2fastq2 Conversion Software (Illumina). Alignment was performed on University of California Santa Cruz human genome reference build 19 using Burrows-Wheeler Aligner. Genome Analysis Toolkit (GATK) and PICARD tools were used for base quality score recalibration (BaseRecalibrator) and realignment (RealignerTargetCreator, IndelRealigner), as recommended by Eurogenetest guidelines (Matthijs et al., 2016). Variant calling was performed using GATK HaplotypeCaller and annotated using EnsemblVariantEffectPredictor. Variants were filtered for quality score ≥ 30, depth ≥ 20x, and present in ≥ 20% of reads.

#### Biological Filters

Because of familial presentation and to identify variants of interest involved in monogenic hereditary cancer predisposition with high penetrance, biological filters included: minor allele frequency < 1% or unknown as determined by Exome Aggregation Consortium and Exome Sequencing Project, truncating variants (nonsense, frameshift), and splice-site variants with significantly modified Human Splice Finder (HSF) score. Synonymous and missense variants were excluded, except for those with an impact on splice-sites. Analysis of copy number variations was not performed. Genes presenting truncating variants identified by WES in our laboratory in at least 20 persons, including healthy individuals and patients with distinct phenotypes, were excluded.

All types of variants present in genes known to be involved in hereditary predisposition to cancer or actionable for other hereditary diseases as defined by the American College of Human Genetics (Green et al., 2013) were systematically analyzed.

#### *In silico* Analysis

Each variant of interest was annotated and interpreted using ALAMUT (Interactive BioSoftware), which includes splice-site analysis tools (HSF) and protein-function prediction tools (SIFT, Polyphen 2.0). Each variant was confirmed by direct reading of sequence using Integrative Genomics Viewer (IGV, Broad Institute). Gene Expression Profiling Interactive Analysis (GEPIA) was used to identify genes of interest due to their expression profiles in papillary thyroid cancer, RC and/or melanoma.

#### Sanger Analysis

Constitutional mutations in genes of interest were confirmed in the proband by Sanger Sequencing, using a 3500 × l instrument and BigDye Terminator kit 3.1 (Applied Biosystems). Primers were designed using PrimerBlast to target variants identified by WES. Primers and experimental conditions are available on request.

Loss of heterozygosity (LOH) was studied for these same variants in the proband’s thyroid tumor samples. LOH was defined by ≥80% reduction of an allele, using Seqman software (DNASTAR).

## Results

Whole-exome-sequencing presented an average depth of 107 reads (107X). Ninety-four percent of the exome was covered at a depth of ≥20X.

The four persons studied presented an average of 30,794 variants meeting quality criteria, of which an average of 2746 were truncating variants. After further bio-informatics filtering, 42 truncating and splice-site variants present in the proband were selected as potentially disease-causing (cf. **Table 2**). These included 18 *de novo* variants of interest, and 24 variants shared with one or the other parent, all of which were heterozygous. The most notable finding was a *de novo* deleterious mutation in *PTEN*: c.1003C > T; p.(Arg335^∗^). Five of the inherited variants in genes associated with cancer or actionable for hereditary diseases have been described in the literature as pathogenic (*MUTYH:* c.933+3A > C) or of unknown significance (*APC*, *APOB*, *DSG2*, *DSP*). Unpublished truncating variants in two genes under-expressed in cancer [*CEACAM1:*c.553_554insAGGC; p.(Leu185Glnfs^∗^26), and *MIB2:* c.153C > A; p.(Cys51^∗^)] were also inherited.

**Table 2:** Variants of interest identified by WES in nuclear family and their segregation.

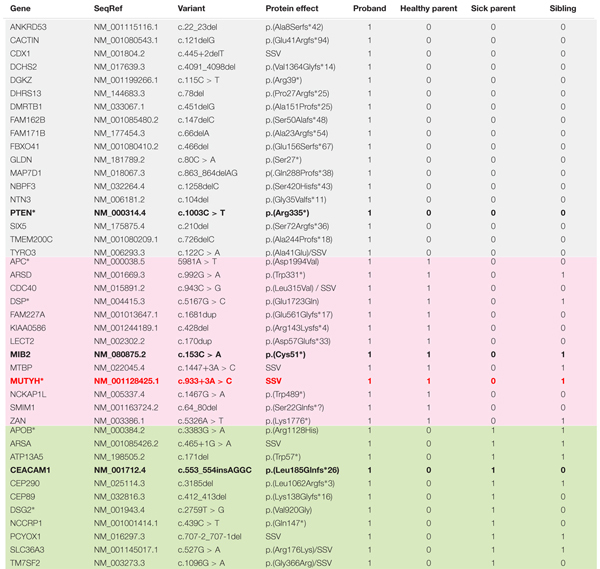

*In gray: 18 *de novo* variants identified in the proband; in pink: variants shared by the proband and healthy parent; in green: variants shared by the proband and affected parent. Black bolded genes were analyzed for LOH. Red bolded gene has confirmed LOH. SSVs, splice site variants, including missense variants. ^∗^Genes systematically analyzed in WES, as recommended by American College of Human Genetics, and including all types of variants.*

The potentially disease-causing variants in *PTEN*, *MUTYH*, *CEACAM1*, and *MIB2* were confirmed in the proband by Sanger sequencing. Tissue samples from the proband’s thyroid tumor were analyzed in parallel for LOH at these sites. LOH was identified in the *MUTYH* gene. No LOH was found in the other genes.

## Discussion

Whole-exome-sequencing of a patient with early onset multiple primary malignant tumors and his nuclear family identified 42 rare truncating and splice-site variants, including a *de novo* deleterious heterozygous mutation in *PTEN*, involved here in atypical presentation of CS.

### Clinical Aspects

With only three minor criteria of CS (follicular variant of papillary thyroid cancer, multinodular goiter, and RC), the patient did not meet diagnostic or testing criteria. However, the head circumference and colonic status were unknown.

Macrocephaly is a frequent feature in *PTEN* mutation carriers, described in 83 to 94% of cases (Mester et al., 2011; Nieuwenhuis et al., 2014). It underlines the importance of cranial perimeter measurement when assessing tumors of the PTEN spectrum. Multiple gastrointestinal intestinal polyps are equally frequent, observed in the colon in up to 93% of cases and the upper gastrointestinal tract in 66–100% of cases, with various histological types (Levi et al., 2011; Stanich et al., 2014). However, macrocephaly and gastrointestinal polyps are not constant, and their absence does not rule out diagnosis of CS.

Our patient had no muco-cutaneous lesions and no evocative upper gastrointestinal lesions. Pathognomonic muco-cutaneous lesions (trichilemmoma, palmoplantar keratosis, oral mucosal papillomatosis, and macular pigmentation of glans penis) are frequent features in CS, with prevalence respectively at 6–38, 82, 85, and 46–53% (Bubien et al., 2013; Pilarski et al., 2013). Lipoma and testicular lipomatosis are described in 30 to 40% (Woodhouse et al., 2005; Gammon et al., 2016). Glycogenic acanthosis and Lhermitte–Duclos disease are present respectively in 48–80 and 2–15% of cases (Levi et al., 2011; Bubien et al., 2013; Pilarski et al., 2013).

Our case was also atypical in regards to the type of cancers diagnosed and age of occurrence. RC presents a lifetime risk of about 1.6% in the general population (Gupta et al., 2017). It is a minor diagnostic criterion of CS with a lifetime maximum risk estimated at 36% (Tan et al., 2012). The prevalence of RC in *PTEN* mutation carriers is low (1.7 to 4%) in large cohort studies (Pilarski et al., 2011; Mester et al., 2012; Tan et al., 2012; Bubien et al., 2013). It is predominantly papillary (I and II) and chromophobe types, unilateral, and occurs around 40–50 years old. One case of bilateral chromophobe carcinoma has been described, and one case of clear cell carcinoma in a male at 56 years of age (Mester et al., 2012; Shuch et al., 2013).

Here, we describe for the first time a case of bilateral clear cell carcinoma of early onset (between 31 and 33 years of age). These tumors are associated with early diagnosis and good prognosis, which is consistent with the data from the literature (Mester et al., 2012; Shuch et al., 2013). However, the diagnosis was made fortuitously. Current guidelines recommend screening for RC using ultrasound every 1–2 years, according to familial history of RC, starting at age of 40 years of age (Saslow et al., 2012). This screening should be started at age of 30 years of age, as proposed by the French Cowden Disease Network (Bubien et al., 2013), for all patients with constitutional deleterious mutation in the *PTEN* gene. As RC occurs frequently without familial history of RC, the periodicity of screening for this cancer should not be based on familial history but be proposed annually.

Other genes predispose to RC, including *VHL*, *MET*, *FH*, *FLCN*, *BAP1*, and *SDHB* (Haas and Nathanson, 2014). Thus, *PTEN* could be included in panel testing of patients with early onset RC, whatever the histological type.

Melanoma presents a lifetime risk of 2.4% in the general population (Apalla et al., 2017). Lentiginous acral melanoma represents 1.5% of melanomas, and is associated with poorer prognosis because of later diagnosis. Most cases appear in the seventh decade on non-sun-exposed sites, which suggested mechanisms of carcinogenesis other than ultraviolet exposure, including genetic factors (Carrera and Puig-Butille, 2018). Lifetime risk is estimated at 6% in *PTEN* mutation carriers and guidelines do not currently recommend systematic dermatological screening in this population (Saslow et al., 2012). Despite the low risk, annual dermatological screening could be considered for all *PTEN* mutation carriers. Because pediatric onset has been described, screening could begin upon diagnosis.

Thyroid carcinoma presents a lifetime risk of about 1% in the general population (Nguyen et al., 2015). In *PTEN* mutation carriers, lifetime risk is estimated at 14 to 38%, with onset in the third decade (Tan et al., 2012; Bubien et al., 2013). Histological sub-types associated with CS include papillary carcinoma (52–60%), follicular carcinoma (14–45%) and follicular variant of papillary carcinoma (4.8–28%) (Laury et al., 2011; Milas et al., 2012). Thyroid carcinoma in our patient is consistent with the data from the literature. He also presented multinodular goiter associated with Hashimoto’s thyroiditis, which is common in case of deleterious mutation in *PTEN*, about 50% (Pilarski et al., 2013).

The risk of a second malignant neoplasm is higher in *PTEN* mutation carriers compared to the general population. From a population of 114 patients with deleterious *PTEN* mutations followed-up during 7 years, a second primary cancer was observed in 40% of cases, with a median interval of 5 years and median age at diagnosis of 50 years. A shorter interval was mostly associated with breast cancer (primary or secondary) (Tan et al., 2012). Of 59 *PTEN* mutation carriers, 20% had two primary cancers, and 5% had three cancers (Bubien et al., 2013). No patient presenting 4 primary tumors has been described in the literature.

Here, we describe for the first time a patient who developed four primary cancers over a period of 2 years. Obesity, the risk of which is increased by *PTEN* haploinsufficiency, could have contributed to the development of RC, which increases by 24 to 34% for every 5 kg/m^2^ rise in BMI (Chow et al., 2010; Pal et al., 2012). However, the change in risk remains low. Another hypothesis is the presence of genetic modifiers. In any case, *PTEN* mutation carriers should take measures to control BMI and stop smoking.

Non-Hodgkin’s lymphoma (NHL) is a heterogeneous cancer, with a worldwide incidence of 13.2 cases for 100,000 persons (Skrabek et al., 2013). The proband’s parent developed two rare forms in the NHL spectrum: orbital lymphoma and small intestine MALT lymphoma, representing respectively 1 and 1.60% of NHL. Most cases occur from 50 to 60 years old. Risk factors include mainly irradiation and infectious agents (Eckardt et al., 2013; Peng et al., 2015). Age of onset and recurrence of MALT lymphoma in the same patient without any known risk factor could suggest genetic factors.

### Molecular Aspects

*PTEN* is a tumor suppressor gene involved in multiple biological processes of carcinogenesis, including regulation of cell growth, proliferation, angiogenesis, and apoptosis by dysregulation of the PI3K/AKT/mTOR pathway. Currently, therapies targeting this pathway showed benefits *in vivo* and *in vitro*. However, efficacies of treatments are temporary and can induce dysmorphologies if administered in early postnatal period (Tachibana et al., 2018).

*De novo* mutation in *PTEN* is frequent, ranging from 10.7 to 47.6% (Mester and Eng, 2012). The nonsense mutation c.1003C > T identified here is commonly observed in PTHS. Interestingly, no genotype–phenotype relationship has been found for mutations in *PTEN* and risk or severity of cancer in CS. Moreover, c.1003C > T can induce the full spectrum of PTHS, even in the same family (**Table 3**; 12, 14, 26, 40–48). This underlines the importance of genetic background and environmental factors.

**Table 3:** Phenotypes and cancers associated with mutation c.1003C > T (p.Arg335X) in PTEN gene in literature.

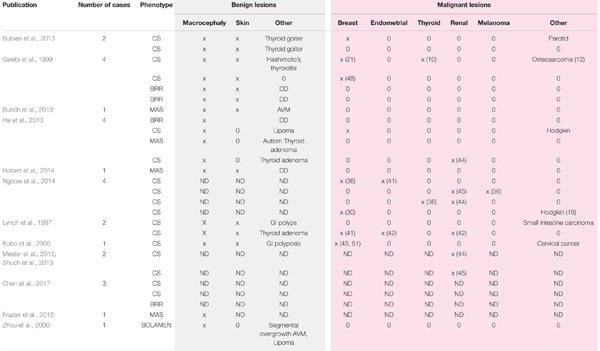

*CS, Cowden syndrome; BRR, Bannayan-Riley-Ruvalcaba syndrome; MAS, Macrocephaly-Autism syndrome; DD, development delay; AVM, arteriovenous malformation; GI, gastrointestinal; x, presence of the item; 0, absence of the item; ND, item not mentioned in publication; number between brackets, age at occurrence of cancer.*

*PTEN* is known to predispose to thyroid carcinoma. We did not observe LOH in the thyroid tumor samples, although this analysis was limited to the site of the mutation. Promotor methylation or acquired mutation elsewhere in the gene could lead to loss of function of *PTEN*, frequent in thyroid carcinoma (Alvarez-Nuñez et al., 2006).

Whole-exome-sequencing of the family made it possible to analyze the familial genetic background. Among the variants observed, two occurred in genes of particular interest: *CEACAM1* and *MIB2*.

*CEACAM1* encodes an immunoglobulin protein involved in cell–cell adhesion expressed in leukocytes, endothelial and epithelial tissues. *CEACAM1* acts as a tumor suppressor gene in the PI3K/AKT pathway in certain epithelial tumors including clear cell RC, while *de novo* expression is a marker of cancer progression in other tumors including melanoma and thyroid carcinoma. Loss of expression was identified in all clear cell RC samples in a case series study, and in renal adenoma, suggesting early involvement in renal carcinogenesis (Kammerer et al., 2004). Thus, truncating mutations in this gene may contribute to the genesis of clear cell RC and could explain in part the early onset clear cell RC observed in the proband. Interestingly, *CEACAM1* is also involved in regulation of lymphocyte B activation and proliferation. CEACAM1-specific monoclonal antibody induces strong B-cell proliferation in mouse, and acts as a negative co-receptor for the B cell receptor, promoting activation-induced cell death in B lymphocytes (Greicius et al., 2003; Lobo et al., 2009). The *CEACAM1* mutation in the proband was inherited from a parent affected with MALT lymphoma, and may contribute to their lymphoproliferative B cell disease, and was absent from the two healthy persons in this study.

A deleterious mutation in *MIB2* was transmitted by the proband’s healthy parent. *MIB2* encodes an E3 ubiquitin protein ligase that mediates ubiquitination of protein in Notch pathways. LOH or promoter hypermethylation of *MIB2* has been associated with melanoma invasion (Takeuchi et al., 2006). A deleterious mutation in this gene could play a role in the development of melanoma in our patient.

The mutation observed in *MUTYH* is known to be pathogenic and involved in MUTYH-associated polyposis, an autosomal recessive disorder. LOH was identified in the proband’s thyroid tumor sample. However, bi-allelic and mono-allelic carriers of MUTYH mutations are not known to be at risk of melanoma, renal or thyroid carcinoma (Kantor et al., 2017).

Four disease–associated genes in the proband presented VUS: *APC*, *APOB*, *DSG2*, and *DSP*, respectively associated with familial adenoma polyposis, homozygous familial hypercholesterolemia and hereditary cardiac disease (Awad et al., 2008; Basso et al., 2017; Defesche et al., 2017). The proband did not have signs of those syndromes.

Atypical presentation of CS described here could suggest systematic annual screening of RC and melanoma in *PTEN* mutation carriers, beginning respectively at 30 years of age and upon diagnosis. Furthermore, *PTEN* could be included in the panel analysis of precocious RC before 50 years, whatever the histological type, even in the absence of muco-cutaneous signs of CS. Prevention of obesity and smoking should be proposed. Heterogeneous phenotypes associated with mutation c.1003C > T p.(Arg335^∗^) suggests the presence of modifier genes. WES allowed analysis of genetic background of nuclear family and identified two candidate modifier genes: *CEACAM1* and *MIB2*. Further studies are required to determine the implication of these genes in the severity of CS in the proband and occurrence of early onset MALT lymphoma in one parent.

## Author Contributions

MC, YB, and Y-JB designed the study. MC, FP-C, and SV performed the acquisition of data. MC, FP-C, MG-B, MP, NU, YB, and Y-JB performed analysis, interpretation of data, and revised critically the article. MC and NU drafted the article.

## Conflict of Interest Statement

The authors declare that the research was conducted in the absence of any commercial or financial relationships that could be construed as a potential conflict of interest.
